# A Naturally Occurring Domestic Cat APOBEC3 Variant Confers Resistance to Feline Immunodeficiency Virus Infection

**DOI:** 10.1128/JVI.02612-15

**Published:** 2015-12-17

**Authors:** Rokusuke Yoshikawa, Taisuke Izumi, Eri Yamada, Yusuke Nakano, Naoko Misawa, Fengrong Ren, Michael A. Carpenter, Terumasa Ikeda, Carsten Münk, Reuben S. Harris, Takayuki Miyazawa, Yoshio Koyanagi, Kei Sato

**Affiliations:** a Laboratory of Viral Pathogenesis, Institute for Virus Research, Kyoto University, Kyoto, Japan; b Department of Microbiology, Institute of Health Biosciences, The University of Tokushima, Tokushima, Japan; c Department of Bioinformatics, Medical Research Institute, Tokyo Medical and Dental University, Tokyo, Japan; d Department of Biochemistry, Molecular Biology and Biophysics, Institute for Molecular Virology, Masonic Cancer Center, and Center for Genome Engineering, University of Minnesota, Minneapolis, Minnesota, USA; e Clinic for Gastroenterology, Hepatology, and Infectiology, Medical Faculty, Heinrich Heine University, Düsseldorf, Germany; f Howard Hughes Medical Institute, University of Minnesota, Minneapolis, Minnesota, USA; g Laboratory of Signal Transduction, Institute for Virus Research, Kyoto University, Kyoto, Japan; h Laboratory of Virolution, Institute for Virus Research, Kyoto University, Kyoto, Japan; i CREST, Japan Science and Technology Agency, Saitama, Japan

## Abstract

Apolipoprotein B mRNA-editing enzyme catalytic polypeptide-like 3 (APOBEC3; A3) DNA cytosine deaminases can be incorporated into progeny virions and inhibit lentiviral replication. On the other hand, viral infectivity factor (Vif) of lentiviruses antagonizes A3-mediated antiviral activities by degrading A3 proteins. It is known that domestic cat (Felis catus) APOBEC3Z3 (A3Z3), the ortholog of human APOBEC3H, potently suppresses the infectivity of *vif*-defective feline immunodeficiency virus (FIV). Although a recent report has shown that domestic cat encodes 7 haplotypes (hap I to hap VII) of A3Z3, the relevance of A3Z3 polymorphism in domestic cats with FIV Vif has not yet been addressed. In this study, we demonstrated that these feline A3Z3 variants suppress *vif*-defective FIV infectivity. We also revealed that codon 65 of feline A3Z3 is a positively selected site and that A3Z3 hap V is subject to positive selection during evolution. It is particularly noteworthy that feline A3Z3 hap V is resistant to FIV Vif-mediated degradation and still inhibits *vif*-proficient viral infection. Moreover, the side chain size, but not the hydrophobicity, of the amino acid at position 65 determines the resistance to FIV Vif-mediated degradation. Furthermore, phylogenetic analyses have led to the inference that feline A3Z3 hap V emerged approximately 60,000 years ago. Taken together, these findings suggest that feline A3Z3 hap V may have been selected for escape from an ancestral FIV. This is the first evidence for an evolutionary “arms race” between the domestic cat and its cognate lentivirus.

**IMPORTANCE** Gene diversity and selective pressure are intriguing topics in the field of evolutionary biology. A direct interaction between a cellular protein and a viral protein can precipitate an evolutionary arms race between host and virus. One example is primate APOBEC3G, which potently restricts the replication of primate lentiviruses (e.g., human immunodeficiency virus type 1 [HIV-1] and simian immunodeficiency virus [SIV]) if its activity is not counteracted by the viral Vif protein. Here we investigate the ability of 7 naturally occurring variants of feline APOBEC3, APOBEC3Z3 (A3Z3), to inhibit FIV replication. Interestingly, one feline A3Z3 variant is dominant, restrictive, and naturally resistant to FIV Vif-mediated degradation. Phylogenetic analyses revealed that the ancestral change that generated this variant could have been caused by positive Darwinian selection, presumably due to an ancestral FIV infection. The experimental-phylogenetic investigation sheds light on the evolutionary history of the domestic cat, which was likely influenced by lentiviral infection.

## INTRODUCTION

During the evolution of viruses and their hosts, cross-species transmission can lead to the emergence of a nascent lineage of viruses (1, 2). On the other hand, intrinsic cellular restriction factors can be barriers that impair cross-species infection and subsequent adaptation of the viruses to new hosts (3). Human apolipoprotein B mRNA-editing enzyme catalytic polypeptide-like 3G (APOBEC3G; A3G), a cytoplasmic DNA cytosine deaminase, is known as a restriction factor that inhibits the replication of human immunodeficiency virus type 1 (HIV-1), the causative agent of AIDS (4). A3G is incorporated into nascent HIV-1 particles and induces G-to-A mutations in the newly synthesized viral cDNA, which results in the abrogation of viral replication. To overcome A3G-mediated restriction, an HIV-1-encoded protein, viral infectivity factor (Vif), impedes A3G packaging into the nascent viral particles by degrading A3G via a ubiquitin/proteasome-dependent pathway (4, 5).

Although rodents have a single *Apobec3* (*A3*) gene, primates, including humans, have 7 distinct *APOBEC3* (*A3*) genes designated *A3A* to *A3H* (4). The seven *A3* genes have been subjected to positive selection during the evolution of primates (6–8). Importantly, Vif counteracts primate A3G in a species-optimized manner (9, 10), suggesting that Vif's specificity reflects the process of adaptation of primate lentiviruses (PLVs) to the hosts. Moreover, the relationship between viral proteins (e.g., Vif) and host proteins (e.g., A3G) can be a clue to elucidate the history of virus-host coevolution and/or their evolutionary “arms race” (6, 11, 12). For instance, *A3G* is highly diversified in Old World monkeys, and the specificity of Vif is dependent on the lineage of PLVs (13, 14). These findings strongly suggest that the diversity of A3 can be a clue to interpretation of past lentivirus infection (13). Moreover, there are at least 7 haplotypes in human A3H; hap II, V, and VII are stable, while the others are unstable (15–17). A subset of A3H haplotypes are resistant to the Vif proteins of some HIV-1 isolates, suggesting that this may be a natural barrier to HIV-1 replication (16, 17).

Feline immunodeficiency virus (FIV), a lentivirus related to HIV-1, was first isolated in 1987 from domestic cats (Felis catus) with chronic AIDS-like disorders (18). Previous studies have shown that domestic cats (Felis catus) encode multiple A3 proteins and that feline A3Z3, the ortholog of human A3H, potently impairs FIV infection through incorporation into the nascent virions (19–22). FIV Vif antagonizes the antiviral activity of feline A3Z3 by degrading this protein (19–22). Because *vif*-deficient FIV is unable to replicate in *in vitro* cell culture systems (23) and *in vivo* experimental models of cats (24), the presence of FIV Vif is a prerequisite for viral replication in the presence of feline A3Z3.

Recently, de Castro et al. conducted a haplotype survey of feline *A3Z3* in 111 domestic cats in Porto Alegre, a city in Brazil, and reported that there are at least seven A3Z3 haplotypes in domestic cats (25). This is reminiscent of the findings determined in HIV-1 Vif and human *A3H* haplotypes (15–17) as described above. However, the functional relevance of FIV Vif to feline *A3Z3* haplotypes is still unclear. In this study, we investigate the antiviral abilities of natural feline A3Z3 variants and consider their relevance to the evolution of the domestic cat. This is the first report to suggest that the relatively recent evolution of the domestic cat may have been influenced by lentivirus infection.

## MATERIALS AND METHODS

### Sequence determination of an FIV *vif* isolate.

RNA was isolated from a virus solution of FIV strain Shizuoka (26) by using a QIAamp viral RNA minikit (Qiagen). Reverse transcription was performed by using SuperScript II reverse transcriptase (Life Technologies), and reverse transcription-PCR was performed by using PrimeSTAR GXL DNA polymerase (TaKaRa) and the primers listed in Table 1. The obtained product was purified by gel extraction and then cloned by using a Zero Blunt TOPO Cloning kit (Life Technologies). The nucleotide sequences were determined by a DNA sequencing service (Fasmac, Kanagawa, Japan), and the data were analyzed by Sequencher v 5.1 software (Gene Codes Corporation). The sequence of FIV (strain Shizuoka) *vif* has been submitted to GenBank.

**TABLE 1:** List of primers used in this study^*a*^

Product	Primer name	Sequence (5′ to 3′)
FIV *vif*	FIV vif Forward	AAAGGATGAAGAGAAGGG
	FIV vif Reverse	CCAGGAGTAAACCCATTT
IMC of *vif*-deleted FIV	Delta Vif Forward	CTGAAGGGGATGAGTGAATAAGATTGGCAGGTAAG
	Delta Vif Reverse	CTTACCTGCCAATCTTATTCACTCATCCCCTTCAG
Firefly luciferase ORF	pTiger/FF luc Forward	TGGCTAGCCACCATGGAAGACGCCAAAAACATAAAG
	pTiger/FF luc Reverse	AGTGGATCCTTACACGGCGATCTTTCCG
Feline A3Z3 E56A	E56A Forward	GAAAAAGCGCCATGCGGCAATGTGCTTTATTGAC
	E56A Reverse	GTCAATAAAGCACATTGCCGCATGGCGCTTTTTC
Feline A3Z3 hap II-HA	A65S Forward	GACAAGATCAAGTCACTGACGCGGG
	A65S Reverse	CCCGCGTCAGTGACTTGATCTTGTC
Feline A3Z3 hap III-HA	A94T Forward	GCCGAGGAACTGGTTACGTTTGTCAAAG
	A94T Reverse	CTTTGACAAACGTAACCAGTTCCTCGGC
Feline A3Z3 hap IV-HA	A65S Forward	GACAAGATCAAGTCACTGACGCGGG
	A65S Reverse	CCCGCGTCAGTGACTTGATCTTGTC
Feline A3Z3 hap V-HA	A65I Forward	GACAAGATCAAGATACTGACGCGGGAC
	A65I Reverse	GTCCCGCGTCAGTATCTTGATCTTGTC
Feline A3Z3 hap VI-HA	V96I Forward	GAACTGGTTGCGTTTATCAAAGACAACCC
	V96I Reverse	GGGTTGTCTTTGATAAACGCAACCAGTTC
Feline A3Z3 hap VII-HA	A65S R68Q Forward	CAAGATCAAGTCACTGACGCAGGACACATC
	A65S R68Q Reverse	GATGTGTCCTGCGTCAGTGACTTGATCTTG
Feline A3Z3 65V-HA	A65V Forward	GCTTTATTGACAAGATCAAGGTGCTGACGCGGGACACA
	A65V Reverse	TGTGTCCCGCGTCAGCACCTTGATCTTGTCAATAAAGC
Feline A3Z3 65L-HA	A65L Forward	GCTTTATTGACAAGATCAAGCTGCTGACGCGGGACACA
	A65L Reverse	TGTGTCCCGCGTCAGCAGCTTGATCTTGTCAATAAAGC
Feline A3Z3 65F-HA	A65F Forward	GCTTTATTGACAAGATCAAGTTCCTGACGCGGGACACA
	A65F Reverse	TGTGTCCCGCGTCAGGAACTTGATCTTGTCAATAAAGC
Feline A3Z3 65G-HA	A65G Forward	GCTTTATTGACAAGATCAAGTTCCTGACGCGGGACACA
	A65G Reverse	TGTGTCCCGCGTCAGGAACTTGATCTTGTCAATAAAGC
Feline A3Z3 65Y-HA	A65Y Forward	GCTTTATTGACAAGATCAAGTACCTGACGCGGGACACA
	A65Y Reverse	TGTGTCCCGCGTCAGGTACTTGATCTTGTCAATAAAGC
MycHis-tagged feline A3Z3	A3 mycHis Forward	NNNNGAATTCGCCACCATGAATCCACTACAG
	A3 mycHis Reverse	NNNNAAGCTTTTCAAGTTTCAAATT
Partial luciferase gene	FF luc Forward	CTRCTRRTRCCAACCCTATTCTCC
	FF luc Reverse	TYAGGYGGTYAAYGATGAAGAAGTG

a pGL3-basic contains a firefly luciferase gene ORF (Promega).

### Plasmid constructions.

The expressing plasmids for C-terminally hemagglutinin (HA)-tagged feline A3Z3 hap II to VII and a series of substitution mutants at position 65 were constructed by using a GeneArt site-directed mutagenesis system (Life Technologies). HA-tagged feline A3Z3 hap I (GenBank accession number EU011792) expression plasmid (based on pcDNA3.1), which was used in our previous paper (27), was used as the template, and the primers listed in Table 1 were used for the construction. Catalytically inactive mutants (E56A substitution) of feline A3Z3 hap I, hap II, and hap V were constructed by using a GeneArt site-directed mutagenesis system. Each wild-type plasmid was used as the template, and the primers used are listed in Table 1. To construct an FIV-based luciferase-expressing reporter plasmid, pTiger-luc, the fragment of the firefly luciferase gene open reading frame (ORF), was inserted into the NheI-BamHI site of pTiger, a third-generation FIV-based lentiviral vector (Addgene catalog no. 1728). To construct a *vif*-deficient infectious molecular clone (IMC) of FIV, a termination trinucleotide (TAA) was inserted into the *vif* ORF of pFIV-14 (FIV IMC strain Petaluma) (28) by using a GeneArt site-directed mutagenesis system (Life Technologies). For an oligonucleotide cleavage assay (see below), mycHis-tagged feline A3Z3 expression plasmid (based on pcDNA3.1-mycHis [Life Technologies]) was constructed by using the primers listed in Table 1. Plasmid integrity was confirmed by DNA sequencing as previously described (27, 29, 30).

### Cell culture.

HEK293T cells (ATCC; CRL-11268), CRFK cells (ATCC, CCL-94), and a HEK293T cell line transduced with the *nls lacZ* gene [293T(LacZ) cells] (31) were cultured in Dulbecco's modified Eagle's medium (Sigma-Aldrich) supplemented with 10% heat-inactivated fetal calf serum and antibiotics (Life Technologies).

### Transfection.

Transfection was performed by using Lipofectamine 2000 (Life Technologies) or PEI Max (GE Healthcare) in accordance with the procedures recommended by the manufacturers. To analyze the anti-FIV activity of feline A3Z3 (see Fig. 2A and B), pFP93 (pFIVgagpolΔ*vif*; a replication-incompetent *vif*-deficient FIV packaging construct derived from clone FIV-34TF10 [GenBank accession number M25381]) (800 ng) (32), pTiger-luc (pFIVΨ-luc) (800 ng), and pMD.G (pVSVg; a vesicular stomatitis virus G [VSVg] expression plasmid) (320 ng) were cotransfected into HEK293T cells (2 × 10^5^ cells) with or without each feline A3Z3 expression plasmid (800 ng) and/or Flag-tagged FIV Vif expression plasmid (1,000 ng) (27). To determine the dose-dependent effect of FIV Vif on feline A3Z3 degradation (see Fig. 2C), pVR1012-FIV Vif-myc (0, 200, or 1,000 ng) (22) and pcDNA3.1-feline A3Z3 hap I or V-HA (0, 200, or 1,000 ng) were cotransfected into HEK293T cells. To analyze the dose-dependent anti-FIV activity of feline A3Z3 and the association with its enzymatic activity (see Fig. 3B and C), pcDNA3.1-feline A3Z3 hap I, II, or V (0, 200, 800, or 1,800 ng) was cotransfected into HEK293T cells together with pFP93, pTiger-luc, and pMD.G. The concentration of the transfected DNA was maintained at a constant level by supplementation with the appropriate vector control.

To analyze the sensitivity of feline A3Z3 to Vif expressed in *cis* from FIV IMC (see Fig. 3E), pFIV-14 or its *vif*-deficient derivative (3,000 ng) was cotransfected into CRFK cells (1.5 × 10^5^ cells) with or without feline A3Z3 expression plasmid (100 ng). To analyze the activity of feline A3Z3 against feline leukemia virus (FeLV) and RD-114 virus (see Fig. 4A and B), p61E (an IMC of FeLV subgroup A) (1,500 ng) (33) or pCRT1 (an IMC of RD-114 virus) (1,500 ng) (34) was cotransfected into 293T(LacZ) cells (2 × 10^5^ cells) with or without each feline A3Z3 expression plasmid (1,500 ng). To analyze the activity of feline A3Z3 against a long interspersed nuclear element-1 (LINE-1) retrotransposition (see Fig. 4C), pL1-enhanced green fluorescent protein (pL1-EGFP; a LINE-1 cell line expressing green fluorescent protein [GFP]) (Addgene catalog no. 42940) (1,500 ng) (35) was cotransfected into HEK293T cells with or without the expression plasmid for each feline A3Z3 or human A3G (500 ng). The LINE-1 retrotransposition assay was performed as previously described (36). To analyze the effect of MG132, a proteasome inhibitor, on feline A3Z3 degradation (see Fig. 5A), each feline A3Z3 expression plasmid (800 ng) was cotransfected with or without the Flag-tagged FIV Vif (1,000 ng) into HEK293T cells (2.5 × 10^5^ cells). Twenty-four hours after transfection, the cells were treated with or without 10 μM MG132 (Sigma) for 16 h prior to harvesting. To analyze the sensitivity of feline A3Z3 mutants to FIV Vif-mediated degradation (see Fig. 5B to D), feline A3Z3 expression plasmids (150 ng) were cotransfected into HEK293T cells (7.5 × 10^4^ cells) with or without Flag-tagged FIV Vif expression plasmid (1,000 ng). In these assays, the cells and supernatants were harvested 2 days after transfection and were used for immunoblot analysis and their respective reporter assays as described below.

### SDS-PAGE/immunoblot analysis.

SDS-PAGE/immunoblot analysis was performed as previously described (29, 30) by using the following antibodies: anti-HA antibody (3F10; Roche); anti-Flag antibody (OctA; Santa Cruz); anti-c-Myc antibody (C3956; Sigma-Aldrich); anti-FIV p27 antibody (PAK3-2C1; Santa Cruz); anti-VSVg antibody (P5DA; Roche); anti-FeLV p24 antibody (PF12J-10A; Santa Cruz); anti-RD-114 virus capsid (CA) antibody (37); and anti-α-tubulin (TUBA) antibody (DM1A [Sigma-Aldrich] or B-5-1-2 [Covance]). For the virus, 500 μl of the virus solution was ultracentrifuged at 100,000 × *g* for 1 h at 4°C using a TL-100 instrument (Beckman), and the pellet was lysed with 1× SDS buffer. For the transfected cells, the cells were lysed with radioimmunoprecipitation assay (RIPA) buffer (50 mM Tris-HCl buffer [pH 7.6], 150 mM NaCl, 1% Nonidet P-40, 0.5% sodium deoxycholate, 0.1% SDS) with protease inhibitor cocktail (Roche).

To calculate the percentage of feline A3Z3 degradation (see percent degradation data in Fig. 5C and D), the band intensities of the immunoblots of feline A3Z3-HA and TUBA were quantified by using Image J software (http://imagej.nih.gov/ij/), and the following formula was used: percent degradation = 100 − {[A3Z3-HA (+Vif)]/[TUBA (+Vif)]}/{[A3Z3-HA (−Vif)]/[TUBA (−Vif)]} × 100.

### Oligonucleotide cleavage assay.

The plasmids expressing feline A3Z3-mycHis haplotypes were transfected into HEK293T cells and feline A3Z3-mycHis proteins were purified from the cell lysates as described previously (38, 39). The single-stranded DNA deaminase assay was performed as described previously (40). Briefly, oligonucleotides were incubated with feline A3Z3-mycHis or control enzymes for 2 h followed by a 5-min incubation with uracil-DNA glycosylase. Reaction products were separated on 15% denaturing gels and scanned on a GE Life Sciences Typhoon FLA-7000 laser scanner. The nucleotide sequences used were as follows: 5′-ATT ATT ATT ATC CCA ATG GAT TTA TTT ATT TAT TTA TTT ATT T-fluorescein (“substrate/product 1”; see Fig. 3D) and 5′-ATT ATT ATT ATT CAA ATG GAT TTA TTT ATT TAT TTA TTT ATT T-fluorescein (“substrate/product 2”; see Fig. 3D).

### FIV reporter assay.

The culture supernatant harvested at 2 days posttransfection was centrifuged and then filtered through a 0.45-μm-pore-size filter (Millipore) to produce a virus solution. The infectivity of the virus solution was measured by luciferase assay. Briefly, 100 μl of the virus solution was inoculated into HEK293T cells in a 96-well plate (Nunc), and the firefly luciferase activity was measured by using a BrillianStar-LT assay system (Toyo-b-net) and a 2030 ARVO X multilabel counter instrument (PerkinElmer) according to the manufacturers' procedures.

### FeLV and RD-114 virus reporter assay.

The culture supernatant harvested at 2 days posttransfection was centrifuged and then filtered through a 0.45-μm-pore-size filter (Millipore) to produce virus solution. The infectivity of virus solution was measured by LacZ marker rescue assay as previously described (31). Briefly, 100 μl of the virus solution with 8 μg/ml Polybrene (hexadimethrine bromide) was inoculated into CRFK cells (for FeLV) or HEK293T cells (for RD-114 virus) in a 96-well plate (Nunc). The β-galactosidase activity was measured by using a Galacto-Star mammalian reporter gene assay system (Applied Biosystems) and a 2030 Arvo X multilabel counter instrument (PerkinElmer) according to the manufacturers' procedures.

### Measurement of G-to-A hypermutation by feline A3Z3.

The efficacy of A3-mediated G-to-A mutation was analyzed as previously described (30, 41). Briefly, the luciferase-expressing FIV-based *vif*-deleted virus was prepared by cotransfection of pFP93, pMD.G, and pTiger-luc with or without feline A3Z3 expression plasmid into HEK293T cells. The culture supernatant at 2 days after transfection was centrifuged and then filtered through a 0.45-μm-pore-size filter (Millipore) to produce virus solution. The virus solution was inoculated into HEK293T cells (0.75 × 10^5^), and the cells were washed with phosphate-buffered saline and harvested at 2 days after infection. DNA was extracted from the cells by the use of a QIAamp DNA blood minikit (Qiagen). To remove the contaminated plasmid DNA, the extracted cellular DNA was treated with DpnI (New England BioLabs). The partial luciferase gene was amplified using PrimeSTAR GXL DNA polymerase (TaKaRa) with the primers listed in Table 1. PCR conditions were as follows: 98°C for 5 min; 30 cycles of 98°C for 10 s, 57°C for 15 s, and 68°C for 30 s; and 68°C for 5 min. The PCR products were cloned into pCR-Blunt II TOPO vector (Life Technologies) and sequenced by a DNA sequencing service (Fasmac). The obtained data were analyzed by using Hypermut 2.0 (http://www.hiv.lanl.gov/content/sequence/HYPERMUT/hypermut.html), and the results were summarized (see Fig. 3A).

### Protein homology modeling.

The three-dimensional (3D) structure model of feline A3Z3 hap I was constructed using the SWISS-MODEL server (42–45) on the basis of the human A3C crystal structure (PDB identifier [ID]: 3VOW) (46). The energy minimization of the 3D structure model constructed by the use of SWISS-MODEL was refined by the ModRefiner algorithm (47). The solvent-accessible surface area was calculated by using the AREAIMOL supported program in the CCP4 program suite (48–50).

### PAML analysis.

Seven A3Z3 sequences were aligned using ClustalW implemented in MEGA6 (51), and the resulting alignment was verified manually at the amino acid level. Then, the phylogenetic tree was reconstructed using the maximum likelihood (ML) method with PhyML (52). The HKY plus Gamma model (53) and 1,000 bootstrap replications were used in the reconstruction. On the basis of the tree, we conducted the analysis for detecting positive selection. First, to determine the characteristics of positive selection across all lineages, two pairs of site models implemented in PAML package v 4.7 (54) were employed for conducting likelihood ratio tests: model 1 (neutral model) versus model 2 (selection model) and model 7 (neutral model assuming a beta distribution) versus model 8 (selection model assuming a beta distribution). Next, since we are particularly interested in testing whether positive selection has operated on those lineages with amino acid changes at codon 65, hap I, hap V, and hap VI, the branch-site model in PAML was further employed. This model allows the ratio of nonsynonymous to synonymous evolutionary changes (the *dN/dS* ratio) to vary among both sites and branches, which enabled us to infer positive selection along a particular lineage or clade (prespecified as foreground branches) (55). In this study, four branches (hap I, hap V, hap VI, and the ancestral branch of hap I and hap V) were specified to be foreground branches. All PAML analyses were carried out using two models of codon usage, F61 and F3x4, and they yielded consistent results. Moreover, the random-effect-likelihood model implemented in the HyPhy package (56) was also employed for detecting positive selection.

### BEAST analysis.

To infer the date of emergence of feline A3Z3 hap V, a Bayesian Markov chain Monte Carlo (MCMC) method, BEAST v 1.7.5 (57), was employed to estimate the time to the most recent common ancestor of hap V. We applied a relaxed molecular clock (ucld) (58) and a constant-size coalescent (59) tree prior to the tree inference. According to a previous study (60), a normally distributed calibration prior with a mean of 131,000 (years ago) and a standard deviation (SD) of 10,000 was used for the root age of the tree. For the evolutionary rate, we used a normal distribution prior as well, with a mean of 0.00215 (per site per million year [Myr]) and a standard deviation of 0.0002 based on the information previously reported (61). Then, three separate MCMC runs with chain lengths of 10 million steps were performed. The convergence for each run was assessed on the basis of the effective sampling size using Tracer v 1.5 (http://tree.bio.ed.ac.uk/software/tracer), and only parameter estimates with an effective sampling size of >200 were accepted for all runs. The maximum clade credibility tree was generated by using TreeAnnotator in the BEAST package after a 10% burn-in, and the final tree was displayed using FigTree v.1.4.2 (http://tree.bio.ed.ac.uk/software/figtree).

### Statistical analyses.

Data were expressed as averages with SD or standard errors of the means (SEM), and significant differences were determined by Student's *t* test. Statistically significant correlation was determined by analysis using the Spearman rank correlation coefficient (*r*_s_).

### Nucleotide sequence accession number.

The sequence of FIV (strain Shizuoka) *vif* has been submitted to DDBJ (accession number LC079040).

## RESULTS

### Positive selection of feline *A3Z3* gene.

**FIG 1:**
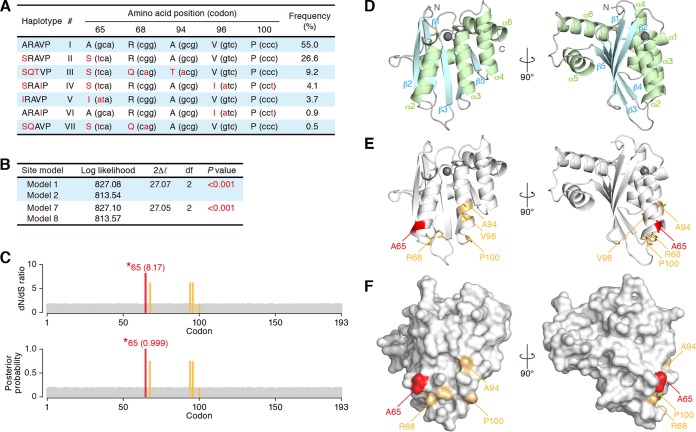
Characteristics of 7 feline A3Z3 haplotypes. (A) Summary of 7 feline A3Z3 haplotypes. Five amino acids and codons (in parentheses) that differed in 7 haplotypes are summarized. The amino acids and nucleotides that differed in the 7 haplotypes are represented in red. Note that the information presented, including the frequency of each haplotype shown here, is from a recent report by de Castro and colleagues, who surveyed 111 domestic cats in Porto Alegre, the capital city of Rio Grande do Sul state in Brazil (25). Also note that the feline A3Z3 used in the previous studies was hap I (GenBank accession number EU011792). (B and C) Positive selection on feline *A3Z3*. (B) Log-likelihood values and parameter estimates determined for two pairs of site models in the PAML package. Positive selection was detected to operate on feline *A3Z3* at a high significance level (*P* < 0.001) by both analyses. 2Δ*l*, twice the log likelihood difference between the compared models; df, degrees of freedom. (C) Identification of codons under conditions of positive selection. The *dN/dS* ratio (top) and posterior probability (bottom) inferred for each codon position by PAML analysis are shown. The positively selected codon identified with high probability (posterior probability, >0.95) is represented in red. The values in parentheses indicate the *dN/dS* ratio (top) and posterior probability (bottom) for the positively selected codon. The bars shown in orange indicate the codons that differed among the 7 haplotypes (codons 68, 94, 96, and 100). (D to F) Structure modeling of feline A3Z3. Cartoon (D and E) and surface (F) models of the structure of feline A3Z3 hap I are shown. In panel D, the α-helix and β-sheet are shown in green and pale blue, respectively. In panels D and E, Zn^2+^ is represented as a gray sphere. In panels E and F, the positively selected amino acid (residue 65) and the amino acids that differed among the 7 haplotypes (residues 68, 94, 96, and 100) are represented in red and orange, respectively. Note that residue 96 (V) is not localized on the surface of a protein.

Here we designate the reported 7 feline *A3Z3* haplotypes (25) hap I to hap VII (Fig. 1A). Four nonsynonymous polymorphisms were detected at codons 65, 68, 94, and 96, and a synonymous substitution was observed at codon 100 (Fig. 1A). In order to infer the molecular evolution of feline *A3Z3*, we performed phylogenetic analyses. As shown in Fig. 1B, we found that feline *A3Z3* was maintained under conditions of positive selection with statistical significance. Moreover, the nonsynonymous-to-synonymous rate (*dN/dS* ratio) at codon 65 was greater than 1 with posterior probability greater than 0.95 (Fig. 1C) (*dN/dS* ratio, 8.17; posterior probability, 0.99). Codon 65 was detected to be maintained under conditions of positive selection by using the random-effect-likelihood analysis as well, with a Bayes factor greater than 50 (*dN*/*dS* ratio, 346.7). These findings indicate that feline *A3Z3* had evolved under conditions of positive selection and, particularly, that codon 65 had been a positively selected site during the evolution.

To assess the position of residue 65 in the tertiary structure of feline A3Z3, we constructed a homology model of feline A3Z3 hap I based on the human A3C crystal structure (46) (Fig. 1D). Residue 65 maintained under conditions of positive selection was located in α-helix 2 (Fig. 1E). Also, 4 of the 5 polymorphic amino acids (65, 68, 94, and 100) localized on the same surface of protein (Fig. 1F).

### Resistance of feline A3Z3 hap V to FIV Vif-mediated degradation.

**FIG 2:**
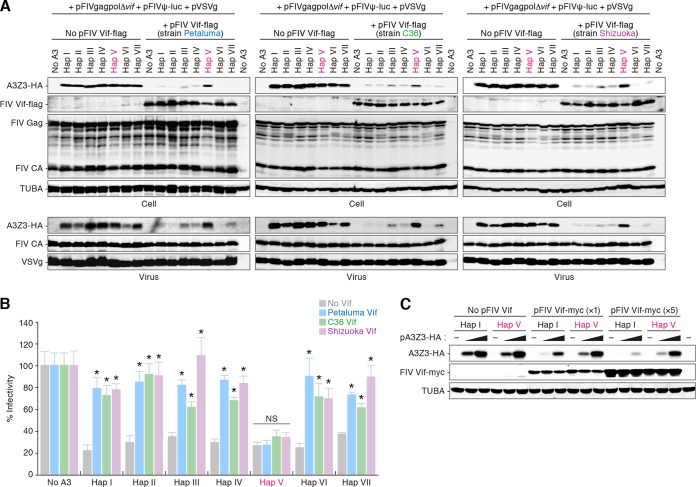
Resistance of feline A3Z3 hap V to FIV Vif-dependent degradation. (A and B) Anti-FIV ability of feline A3Z3 haplotypes. (A) Immunoblot analysis. Representative results for FIV Vif strains Petaluma (top), C36 (middle), and Shizuoka (bottom) are shown. (B) FIV reporter assay. FIV infectivity is shown as the percentage of the value of “no A3.” (C) Correlation of the expression levels of FIV Vif and feline A3Z3. Data are representative of the results of immunoblot analysis. In panel B, asterisks (*) represent a *P* value of <0.05 (versus “no Vif” by Student's *t* test). NS, no statistical significance. The assays were independently performed in triplicate. Data represent averages with SD.

To elucidate the relationship of feline A3Z3 polymorphism with FIV infection, we investigated the anti-FIV activity of the 7 A3Z3 haplotypes. In the absence of FIV Vif, the expression levels of these 7 feline A3Z3 haplotypes were similar, and the incorporation efficacies of these A3Z3 proteins into FIV particles were comparable (Fig. 2A). In addition, the infectivity of *vif*-deleted FIV was significantly suppressed by all feline A3Z3 haplotypes at comparable magnitudes (Fig. 2B). We then assessed the activity of feline A3Z3 haplotypes in the presence of FIV Vif. Expression plasmids were prepared for FIV Vif of 3 strains: Petaluma (28), C36 (62), and Shizuoka (26). In particular, to be able to use the FIV *vif* sequence of a clinical isolate, we determined the *vif* sequence of FIV strain Shizuoka (GenBank accession number LC079040). As shown in Fig. 2A, it was of interest that all 3 strains of FIV Vif proteins were unable to degrade A3Z3 hap V, while the other A3Z3 haplotypes were degraded in an FIV Vif-dependent manner (Fig. 2A). Moreover, even in the presence of Vif, A3Z3 hap V was efficiently packaged into the released virions (Fig. 2A) and significantly suppressed FIV infectivity (Fig. 2B). To ask whether the ratio of the expression levels of FIV Vif to those of feline A3Z3 affects this observation, we cotransfected the expression plasmids for FIV Vif (strain Petaluma) and feline A3Z3 at 3 different doses, respectively. As shown in Fig. 2C, the resistance of feline A3Z3 hap V to FIV Vif-dependent degradation was maintained regardless of the expression levels of feline A3Z3 and FIV Vif. These findings suggest that feline A3Z3 hap V is resistant to FIV Vif-mediated degradation and elicits anti-FIV ability even in the presence of FIV Vif.

**FIG 3:**
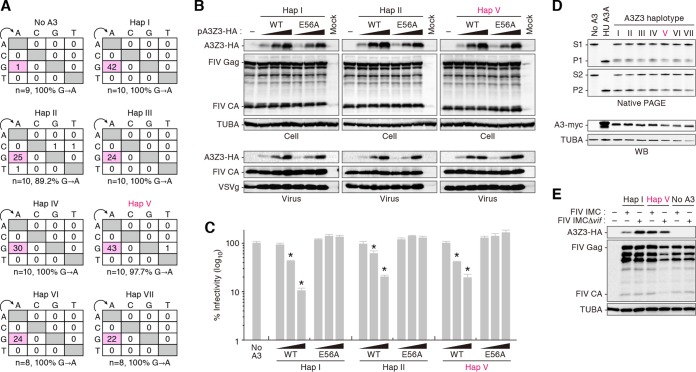
Deaminase-dependent anti-FIV ability of feline A3Z3 haplotypes. (A) Mutations in *vif*-deficient FIV-based reporter provirus. The partial luciferase gene (411 bp) was cloned and sequenced, and the results are summarized in the mutation matrix. The numbers of amplicons sequenced (n) and the percentages of G-to-A mutations in all substitutions are represented. (B and C) Dose and deaminase-dependent anti-FIV ability of feline A3Z3. (B) Representative results of immunoblot analysis. WT, wild type. (C) FIV reporter assay. FIV infectivity is shown as the percentage of the value corresponding to “no A3.” (D) Enzymatic activity of feline A3Z3 haplotypes. Representative results of oligonucleotide cleavage assay (top) and immunoblot analysis (bottom) are shown. In the top panel, the enzymatic activity of feline A3Z3 haplotypes in the 2 different contexts (substrate/product 1 and product 2) was measured. Human (HU) A3A tagged with mycHis was used as a positive control in the assay. S, substrate; P, product; WB, Western blot. (E) Sensitivity of feline A3Z3 hap I and hap V to a FIV IMC in feline CRFK cells. Representative results of immunoblot analysis are shown. In panel C, an asterisk (*) represents *P* < 0.05 (versus “no A3” by Student's *t* test). The assays were independently performed in triplicate. Data represent averages with SD.

Next, we addressed whether the deaminase activity of feline A3Z3 is associated with its anti-FIV ability. In line with previous reports (19, 20), feline A3Z3 hap I induced a G-to-A hypermutation in a *vif*-deficient FIV-based reporter virus (Fig. 3A). The other haplotypes, including hap V, also induced G-to-A mutations (Fig. 3A). In addition, we prepared catalytically inactive (E56A) derivatives of feline A3Z3 hap I, II, and V. As shown in Fig. 3B and C, wild-type feline A3Z3 proteins were incorporated into the released virions and suppressed FIV infectivity in dose-dependent manners. Although the E56A derivatives were packaged into the virions similarly to wild-type A3Z3 (Fig. 3B), these enzymatically inactive mutants were incapable of suppressing FIV infectivity (Fig. 3C). These findings strongly suggest that feline A3Z3 impairs FIV infection dependently of its catalytic activity. Furthermore, to assess the enzymatic activity of the respective feline A3Z3 haplotypes, we performed single-stranded DNA deamination assays, which measure the biochemical activity of A3 proteins *in vitro* (40). As shown in Fig. 3D, all feline A3Z3 haplotypes exhibited enzymatic activity at comparable levels. Taken together, these findings suggest that feline A3Z3 suppresses FIV infectivity in a deaminase-dependent manner and that the feline A3Z3 polymorphisms described here do not affect the enzymatic activity.

To assess the relevance of these findings (Fig. 2 and 3A to D) under more physiologically relevant conditions, the expression plasmids for A3Z3 hap I or hap V were cotransfected with an IMC of FIV or its *vif*-deleted derivative into feline CRFK cells. As shown in Fig. 3E, feline A3Z3 hap V was resistant to the degradation mediated by Vif, which was expressed in *cis* from FIV IMC. These data further suggest that feline A3Z3 hap V is resistant to FIV Vif-mediated degradation.

### Resistance of feline gammaretroviruses and retrotransposons to feline A3Z3-mediated suppressive activity.

**FIG 4:**
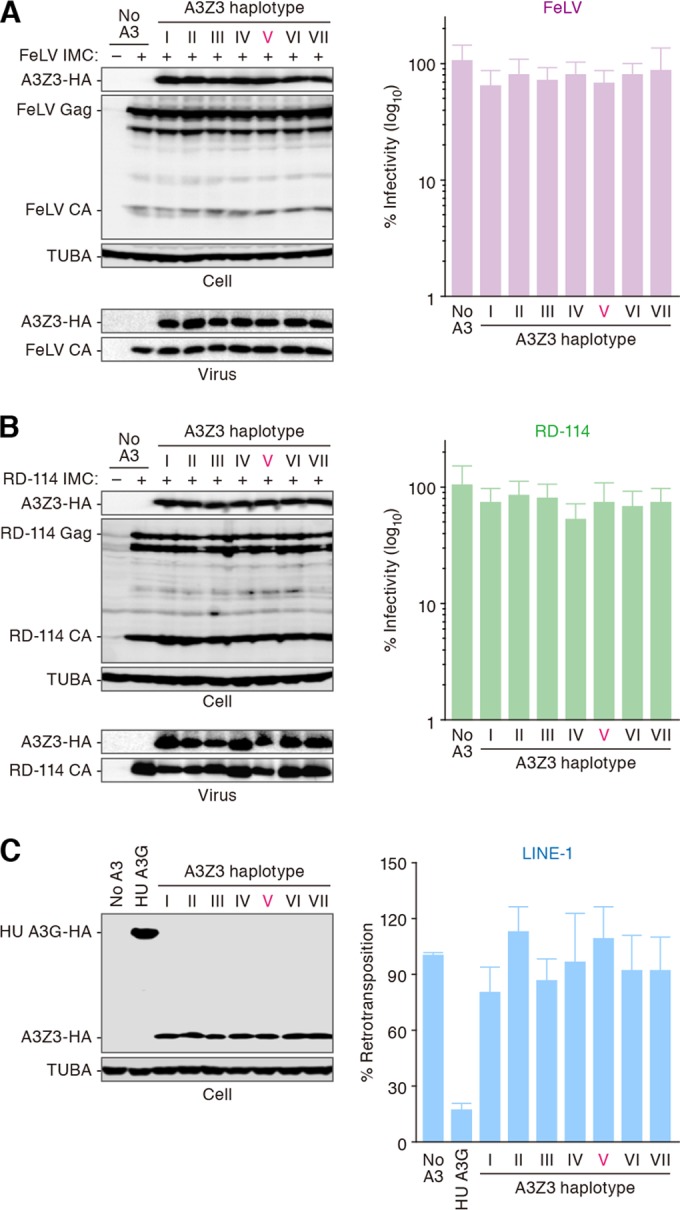
The effect of feline A3Z3 haplotypes against FeLV, RD-114 virus, and a retrotransposon. FeLV infectivity (A), RD-114 virus infectivity (B), and LINE-1 retrotransposition (C) are shown as percentages of the value corresponding to “no A3.” Representative results of immunoblot analysis are shown in the left panels. In the right panels, each value is shown as the percentage of the value corresponding to “no A3.” The assays were independently performed in triplicate. Data represent averages with SD.

To seek the possible relevance of feline A3Z3 haplotypes to the infectious agents other than FIV, we assessed the inhibitory activity of feline A3Z3 with respect to feline leukemia virus (FeLV), a feline endogenous retrovirus (RD-114 virus), and a non-long terminal repeat (LTR) retrotransposon, long interspersed nuclear element-1 (LINE-1). As shown in Fig. 4A and B, all feline A3Z3 haplotypes were equally expressed in the presence of FeLV and RD-114 virus at comparable levels and were incorporated into the released particles of FeLV and RD-114 virus. Nevertheless, the infectivity of FeLV and RD-114 virus was not suppressed by the feline A3Z3 proteins (Fig. 4A and B). Moreover, although human A3G impaired the retrotransposition of LINE-1, which is concordant with a previous report (36, 63), all feline A3Z3 haplotypes did not (Fig. 4C). Taken together, these results suggest that feline A3Z3 polymorphism is not crucially associated with FeLV, RD-114 virus, and L1 replication and that these parasites are resistant to feline A3Z3-mediated suppressive activity.

### Determinant responsible for the sensitivity of feline A3Z3 to FIV Vif-mediated degradation.

**FIG 5:**
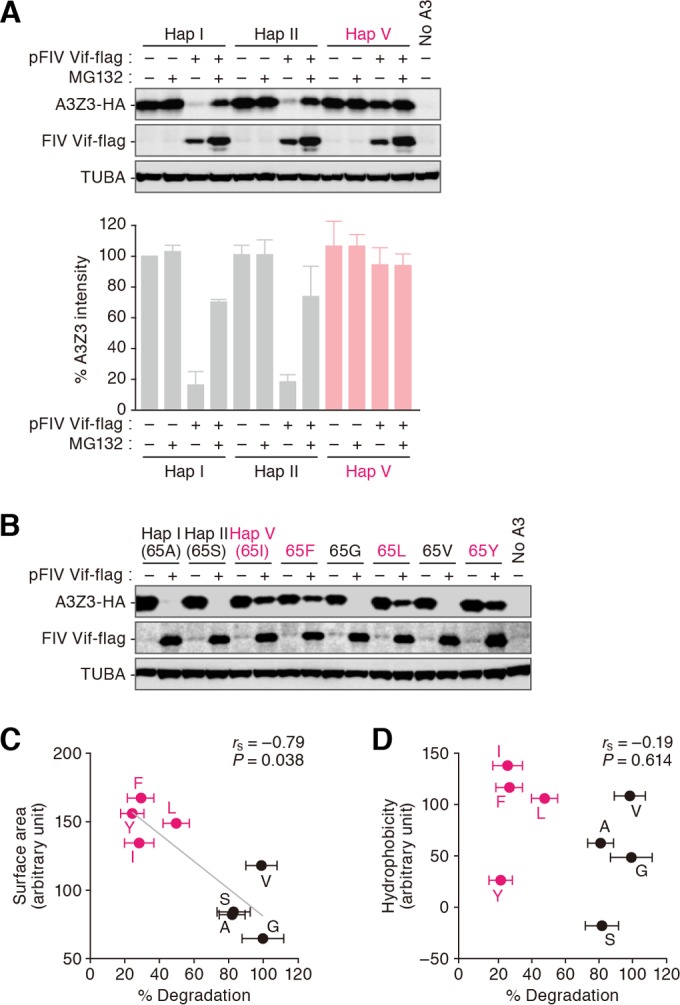
Determinant of feline A3Z3 sensitivity to FIV Vif-mediated degradation. (A) Proteasome-dependent degradation of feline A3Z3 by FIV Vif. The transfected cells were treated with or without 10 μM MG132. Representative results of immunoblot analysis are shown in the top panel, and percentages of the band intensity of A3Z3 compared to hap I without FIV Vif-Flag and MG132 are shown in the bottom panel. This assay was independently performed in triplicate. Data represent averages with SD. (B to D) Determinant responsible for the resistance of feline A3Z3 to FIV Vif-mediated degradation. (B) A representative result of immunoblot analysis. (C and D) The correlations of the percentage of the degradation efficacy of each feline A3Z3 mutant (*x* axes) with the surface area (*y* axis in panel C) or the hydrophobicity (*y* axis in panel D) of residue 65 are shown. The capitalized characters represent the amino acid at position 65. The variants that were resistant and sensitive to FIV Vif are represented in red and black, respectively. The Spearman rank correlation coefficient (*r*_s_) was used to determine statistically significant correlations between the values. The assay was independently performed in triplicate. Data represent averages with SEM. In panel C, the line represents a linear approximation.

To address whether the resistance of feline A3Z3 hap V to FIV Vif-mediated degradation is related to proteasome-dependent protein degradation, the cells expressing feline A3Z3 were treated with MG132, a proteasome inhibitor. As shown in Fig. 5A, MG132 treatment abrogated FIV Vif-mediated degradation of A3Z3 hap I and hap II, suggesting that these A3Z3 are degraded via proteasome. However, the expression level of A3Z3 hap V in MG132-untreated cells was comparable to that in MG132-treated cells (Fig. 5A). These data suggest that FIV Vif degrades feline A3Z3 hap I and hap II but not hap V via proteasome-dependent pathway.

Because the presence of amino acid 65 is the only factor that distinguishes A3Z3 hap V from other haplotypes (Fig. 1A), the resistance of hap V to FIV Vif-mediated degradation is most likely determined by this residue. To test this, we constructed derivatives of hap I in which residue 65 was replaced. These amino acid substitution mutants were expressed similarly in the absence of FIV Vif (Fig. 5B). However, 65F, 65L, and 65Y mutants as well as a 65I (hap V) mutant were resistant to Vif-mediated degradation, whereas 65G and 65V mutants as well as 65A (hap I) and 65S (hap II) mutants were sensitive (Fig. 5B). We further investigated the determinant of this resistance and detected a statistically significant correlation between the exposed surface size at position 65 and the degradation efficacy (Fig. 5C) (*P* = 0.038; *r*_s_ = −0.79). However, there was no correlation between the hydrophobicity and the degradation efficacy (Fig. 5D). Taken together, these results suggest that the exposed outside surface area of the side chain of residue 65, but not hydrophobicity, determines the sensitivity to FIV Vif-mediated degradation.

### Emergence of feline A3Z3 hap V during the late Pleistocene period.

**FIG 6:**
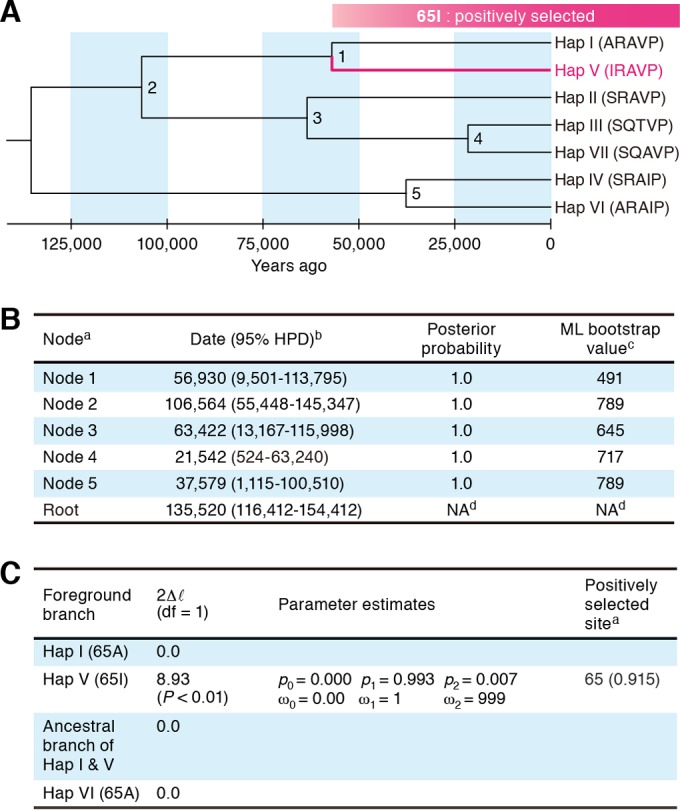
Estimation of the age of feline A3Z3 hap V emergence. (A) Maximum clade credibility tree inferred by using BEAST v 1.7.5. Asterisks indicate the nodes with a posterior probability value of >0.5. The lineage detected under conditions of positive selection by the branch-site test (see panel C) is represented in red. (B) Divergence times and posterior probabilities inferred by BEAST. In the column with the heading with the superscript “a,” node numbers are consistent with the numbers in panel A. In the column with the heading with the superscript “b,” HPD represents “highest posterior density” and the units in the column represent years. In the column with the heading with the superscript “c,” the ML bootstrap value is for 1,000 bootstrap replicates. In the entries with the superscript “d,” NA represents “not applicable.” (C) Likelihood ratio test statistics of 7 feline A3Z3 haplotypes under the branch-site models in PAML analysis. In the column with the heading with the superscript “a,” the number in parentheses represents posterior probability. df, degrees of freedom.

It has been previously reported that a wildcat lineage (Felis silvestris) that included domestic cat diverged around 131,000 years ago (60). On the basis of this information and the evolutionary rate of *A3* genes in mammals (61), we estimated the emergence era of feline A3Z3 hap V using BEAST (57). As shown in Fig. 6A and B, hap V was inferred to have diverged from the common ancestor of hap I and hap V around 60,000 years ago (node 1 in Fig. 6B). In addition, we inferred from the branch-site model that the hap V lineage was maintained under conditions of positive selection (Fig. 6C), whereas no positive selection was detected for hap I, hap VI, and the ancestral branch of hap I and hap V (Fig. 6C). Taken together, these results suggest that feline A3Z3 hap V (65I) emerged under conditions of positive selection around 60,000 years ago.

## DISCUSSION

We inferred from phylogenetic analyses that codon 65 of feline A3Z3 is a positively selected site (Fig. 1B and C) and that hap V has been evolving under conditions of positive selection (Fig. 6C). These results indicate that isoleucine at position 65 in feline A3Z3 has been positively selected during the evolution of domestic cats (Fig. 6A). Importantly, we found that the common ancestral sequence of feline A3Z3 is identical to that of A3Z3 hap VI (data not shown). Because A3Z3 hap VI is sensitive to FIV Vif-mediated degradation (Fig. 2A), our results indicate that ancestral feline A3Z3 is sensitive to FIV Vif and further suggest that the resistance of feline A3Z3 hap V to FIV Vif may have been acquired during the evolution of domestic cats. Importantly, the feline A3Z3 polymorphism failed to associate with infectious agents other than FIV such as FeLV and RD-114 virus (Fig. 4), and feline A3Z3 hap V was resistant to Vif proteins of various FIV strains (Fig. 2). These findings provide evidence suggesting that positive selection pressure has operated on feline A3Z3 hap V and that the positive selection pressure was probably triggered by an ancestral FIV or FIV-related virus. Interestingly, our findings suggest that the gain-of-function mutation in feline A3Z3 was triggered by positive Darwinian selection acting on a lineage of domestic cats (hap V). In comparison, the reported loss-of-function mutation of human A3H most likely occurred after the divergence of the human and chimpanzee lineages (15). Taken together, these results suggest that the evolutionary relationships between hosts and lentiviruses have the potential to be different in each relevant mammalian species.

A large-scale investigation revealed that a lineage of wildcats (Felis silvestris) that included domestic cat split from other wildcat lineages around 131,000 years ago (60). By using this information, we estimated here that feline A3Z3 hap V emerged around 60,000 years ago (Fig. 6A and B). In this regard, an archeological report has suggested that the domestication of cat occurred around 10,000 years ago in the Mesopotamian area (64). Taken together with our results, those findings suggest that feline A3Z3 hap V emerged during the late Pleistocene era and was subjected to positive selection before cat domestication. Moreover, our findings suggest that the ancestors of domestic cats had already been infected with ancient FIV or FIV-related virus during that era and that the infection triggered the positive selection pressure on feline *A3Z3*, leading to the emergence of hap V. Furthermore, another previous report suggested that domestic cats originated from wildcats harboring at least 5 mitochondrial DNA haplotypes (60). This further implies that the polymorphism of feline *A3Z3* can be attributed to the origin of various domestic cat lineages. However, we noticed that the estimates for node ages showed a quite wide 95% highest-posterior-density (95% HPD) interval (Fig. 6B). Since only seven sequences were available for the analysis, we speculate that the higher degree of uncertainty for data estimates produced by BEAST analysis was due to the limited number of sequences and/or the smaller amount of information included in the data set. To infer the divergence times more precisely, larger data sets will be needed. Nevertheless, the divergence times estimated in this study would provide useful starting points for further analysis. It should be noted that the feline *A3Z3* haplotypes used here were inferred on the basis of a recent survey by de Castro and colleagues (25). Since their studies were conducted using 111 domestic cats in Porto Alegre, the capital city of Rio Grande do Sul state in Brazil (25), the frequency of feline A3Z3 hap V may be relevant only to this region of Brazil. Therefore, the correlation of hap V frequency with the global biogeography and the breeding history of domestic cats also warrants further investigation.

In the case of human A3H, residue 121 is located on the protein surface and its hydrophobicity determines human A3H sensitivity to HIV-1 Vif-mediated degradation (15, 17, 65). Similarly to the case of human A3H, residue 65 of feline A3Z3, which determines sensitivity to FIV Vif, is located on the surface of protein (Fig. 1F). However, we revealed that feline A3Z3 sensitivity to FIV Vif is determined by the surface size but not the hydrophobicity of residue 65 (Fig. 5B to D). These findings suggest that the manner of resistance of feline A3Z3 hap V to FIV Vif is different from that of human A3H to HIV-1 Vif.

We revealed that two feline gammaretroviruses, FeLV and RD-114 virus, are resistant to the feline A3Z3-mediated antiviral effect whereas feline A3Z3 is incorporated into the nascent virions (Fig. 4A and B). In this regard, a previous paper suggested that viral protease of murine leukemia virus (MLV), a murine gammaretrovirus, degrades murine A3 and prevents its packaging into the released virions (66). However, degradation of feline A3Z3 proteins was not observed in the cells producing FeLV and RD-114 virus, and feline A3Z3 proteins were incorporated into the released virions (Fig. 4A and B). Therefore, these results suggest that the viral protease-mediated pathway which has been observed in MLV and murine A3 (66) is not associated with the escape of FeLV and RD-114 virus from recognition by feline A3Z3. Rather, it has been reported that the glycosylated Gag protein (glycoGag) of MLV limits the accessibility of murine A3 to the viral reverse transcription complex and thereby prevents the murine A3-mediated anti-MLV effect (67). FeLV, RD-114 virus, and MLV belong to the genus gammaretrovirus, and it was previously directly demonstrated that FeLV expresses glycoGag (68). Therefore, it is conceivable that FeLV and RD-114 virus could avoid the recognition by feline A3Z3 through a glycoGag-dependent pathway.

In terms of natural resistance to FIV, hap V may be beneficial for cats expressing this A3Z3 enzyme. However, its frequency is rather low compared to those of other haplotypes (Fig. 1A). Similarly, the frequency of humans with genes encoding stable A3H (hap II, V, and VII) is not dominant in human population despite their robust anti-HIV-1 ability (15–17). In this regard, a previous report has shown that stable A3H proteins potently inhibit the retrotransposition of retroelements such as LINE-1 and MusD (15). Since it has been suggested that LINE-1 retrotransposition is crucial for the development of human neurons (69) and early embryos (70), the inhibition of retrotransposition by stable A3H proteins may be detrimental to humans and, as a result, stable *A3H* alleles might have lost activity during the evolution of humans (15). However, in contrast to human A3H, all examples of feline A3Z3 that included hap V did not exhibit antiretrotransposition ability (Fig. 4), suggesting that feline A3Z3 polymorphism is not attributable to the retrotransposition-dependent toxicity to hosts. Rather, the association of viral infection with *A3* polymorphism is reminiscent of observations concerning Friend MLV (FrMLV) infection in mouse (Mus musculus). Previous studies have revealed that the levels of FrMLV pathogenicity are different among mouse strains (71) and are determined by the expression level of the murine *A3* gene, of which the feline *A3Z3* gene and the human *A3H* gene are orthologs (72). Moreover, it was of interest that there are naturally occurring polymorphisms in the *A3* gene of the genus *Mus* (72, 73) and that the expression level of the murine *A3* gene is influenced by its genotype (72). These findings suggest that the differences in susceptibility to FrMLV infection are determined by *A3* polymorphism and that the *A3* polymorphism in the genus *Mus* might be the result of the evolutionary pressure during the evolution of the genus *Mus* (72). In the case of feline *A3Z3* polymorphism, the prevalence of FIV and/or FeLV infection in the cats with genes encoding *A3Z3* hap V appears to be lower than that in the cats with the other haplotypes (25). However, we demonstrated that not all feline A3Z3 that included hap V exhibited anti-FeLV ability (Fig. 4A). In contrast, all feline A3Z3 showed robust anti-FIV ability, and, in particular, only hap V is resistant to FIV Vif-mediated degradation (Fig. 2). Taken together, these findings strongly suggest that feline A3Z3 polymorphism is closely associated with the infection of FIV rather than FeLV in domestic cats.

In summary, we demonstrated that feline A3Z3 hap V is maintained under conditions of positive selection and is resistant to FIV Vif-dependent degradation. Although the evolutionary arms race between primate A3 and PLV Vif has been rigorously investigated (6, 11–13, 15, 74), there have been no studies comparing the evolutionary dynamics of nonprimate A3 and nonprimate lentiviruses. To the best of our knowledge, this is the first report providing evidence of the evolutionary arms race between domestic cat and its lentivirus FIV.
